# Poly(amidoamine) Dendrimer as an Interfacial Dipole Modification in Crystalline Silicon Solar Cells

**DOI:** 10.1021/acs.jpclett.3c00643

**Published:** 2023-05-03

**Authors:** Thomas Tom, Eloi Ros, Julian López-Vidrier, José Miguel Asensi, Pablo Ortega, Joaquim Puigdollers, Joan Bertomeu, Cristobal Voz

**Affiliations:** ‡ Departament de Física Aplicada, Universitat de Barcelona (UB), 08028 Barcelona, Spain; § Institute of Nanoscience and Nanotechnology (IN2UB), 08028 Barcelona, Spain; ∥ Departament d’Enginyeria Electrònica, Universitat Politècnica de Catalunya (UPC), 08034 Barcelona, Spain

## Abstract

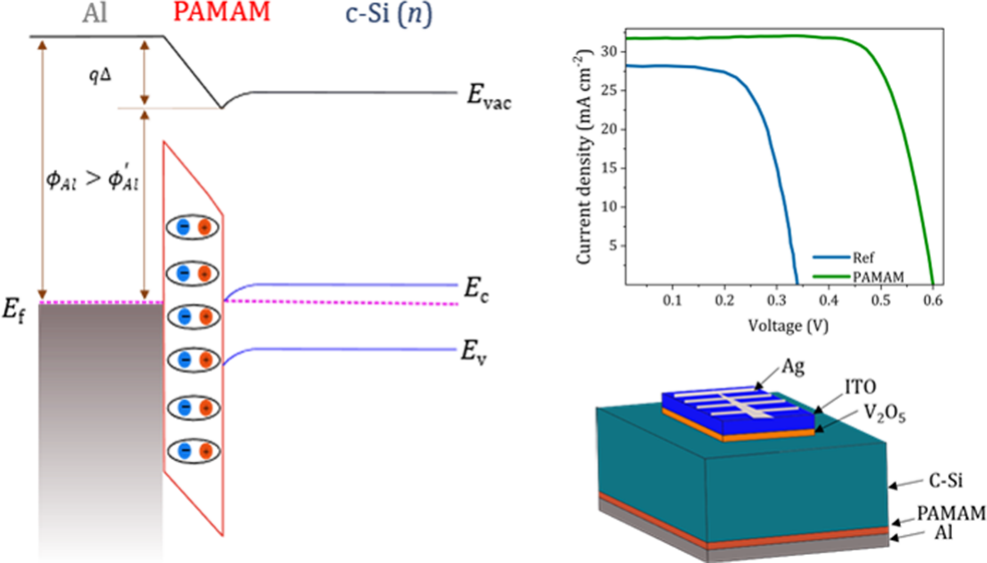

Poly(amidoamine) (PAMAM) dendrimers are used to modify the interface of metal–semiconductor junctions. The large number of protonated amines contributes to the formation of a dipole layer, which finally serves to form electron-selective contacts in silicon heterojunction solar cells. By modification of the work function of the contacts, the addition of the PAMAM dendrimer interlayer quenches Fermi level pinning, thus creating an ohmic contact between the metal and the semiconductor. This is supported by the observation of a low contact resistivity of 4.5 mΩ cm^2^, the shift in work function, and the n-type behavior of PAMAM dendrimer films on the surface of crystalline silicon. A silicon heterojunction solar cell containing the PAMAM dendrimer interlayer is presented, which achieved a power conversion efficiency of 14.5%, an increase of 8.3% over the reference device without the dipole interlayer.

The development of carrier-selective contacts in solar cells is of uttermost interest in the photovoltaic field, which seeks the improvement of the power conversion efficiency (PCE) of heterojunction silicon solar cells.^1,2^ In recent years, researchers have been trying to replace the heavily doped hydrogenated amorphous silicon films that are used as carrier-selective contacts as a result of the complicated fabrication procedure that they require, their parasitic absorption, and their high cost. With this objective in mind, various metal oxides, fluorides, nitrides, and organic molecules have been explored.^3−6^ Among the latter, solution-processable organic semiconductor molecules have gained more acceptance as a result of their simple, low-cost, and low-temperature fabrication process based on the spin-coating technique.

Organic semiconductors are currently being widely investigated as a result of their specific features, like low-production cost, limited processing time, lightweightness, and mechanical flexibility. They are utilized in visible lasers, light-emitting diodes, solar cells, and optical amplifiers.^7−10^ Dendrimers are a new class of organic semiconductors that are well-defined multivalent non-dispersed macromolecules. Dendrimers show some advantages over other organic compounds as a result of better solubility determined by their functional groups, nanoscaled size, and low viscosity.^11−14^ Dendrimers have been studied in the past decade as an emerging material in photovoltaics. Mozer et al. investigated the charge transfer properties of different thiophene dendrimers in a fullerene bulk heterojunction, achieving a PCE of 0.72%.^15^ Highly efficient inverted polymer solar cells with a solution-processable dendrimer as the electron-collection interlayer was investigated by Murugesan et al., which yielded an efficiency of 3.53%.^16^ The formation of an oriented dipole layer and the resulting Helmholtz potential have been cited as the origin of the conjugated polyelectrolyte working concept.^17^ This potential allows for the modification of energy barriers that are often caused by charge transfer events, like Fermi level pinning at the interface between a semiconductor and a metal.^18^

Here, we report the electrical, optical, morphological, and dipolar characteristics of solution-processed thin films of a poly(amidoamine) (PAMAM) generation zero (G0) dendrimer, whose three-dimensional (3D) molecular structure is shown in Figure 1a. As a proof of concept, the PAMAM dendrimer is used as an electron-selective contact on a dopant-free heterojunction solar cell with vanadium pentoxide (V_2_O_5_) as the hole-selective contact, therefore avoiding the use of expensive deposition systems to grow amorphous silicon layers. The solar cell fabricated using this configuration achieved a PCE of 14.5%, which supposes an 8.3% enhancement with respect to the reference device without the PAMAM dendrimer interlayer.

**Figure 1:**
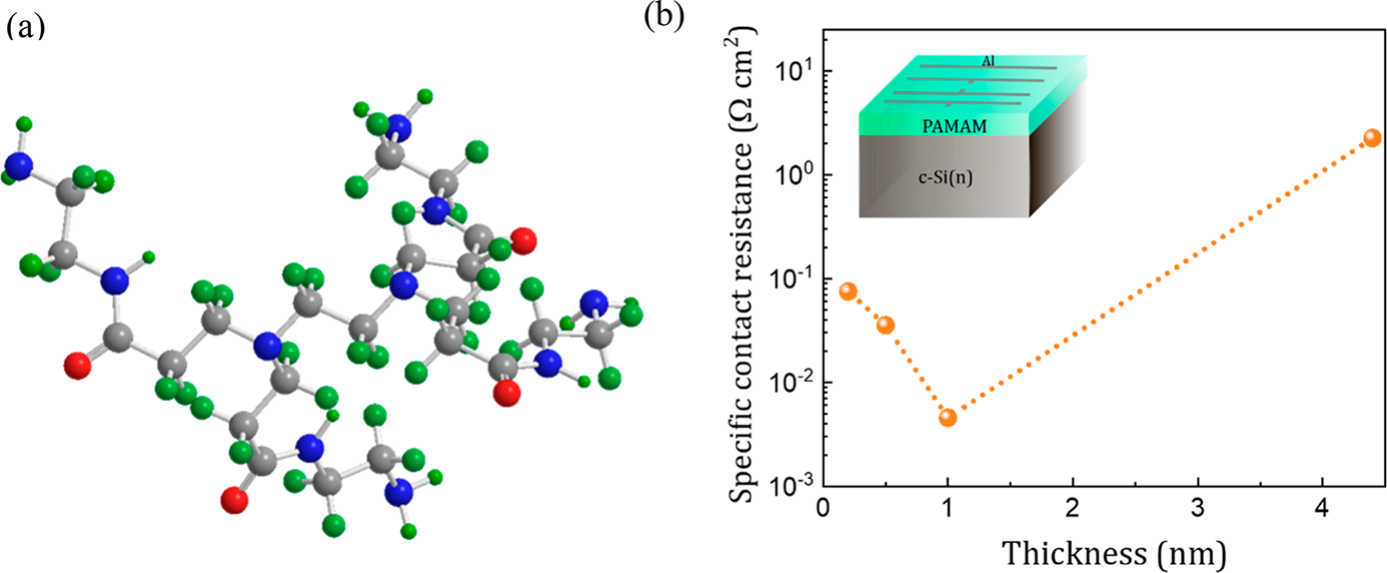
(a) 3D molecular structure of the PAMAM G0 dendrimer: blue, nitrogen; gray, carbon; red, oxygen; and green, hydrogen. (b) Specific contact resistance for varying thickness of the PAMAM dendrimer as extracted from the TLM measurements. The inset displays the TLM contact schematics for the measurements.

The current–voltage (*I*–*V*) characteristics of the PAMAM dendrimer films spin coated on n-type c-Si wafers were studied using the transfer length measurement (TLM) method. The thickness relationship with the contact resistance of the films is plotted in Figure 1b. The inset shows the Al contact structure for TLM characterization. Different concentrations of the solution, from 0.001 to 0.1%, were used, with the concentration as a function of thickness being shown in Figure S1 of the Supporting Information. The optimum contact resistance, of 4.5 mΩ cm^2^, was obtained for the 1 nm thick film with 0.01% concentration. On the one hand, the contact becomes more resistive when increasing the film thickness, suggesting electron tunneling through the PAMAM dendrimer films as the dominating conduction mechanism. On the other hand, the increment in contact resistance below 1 nm indicates at least a secondary mechanism arising at close to monolayer thickness. Therefore, the optimum thickness of 1 nm, at a concentration of 0.1%, was chosen for the following studies.

The surface roughness of 1 nm thick PAMAM dendrimer films on silicon substrates was studied by atomic force microscopy (AFM), and it is shown in Figure 2a. The root-mean-square (RMS) roughness of the film is estimated to be 0.09 nm. The low surface roughness and lack of extended sharp peaks indicates a good uniformity of the PAMAM dendrimer films and enhanced wettability over the Si surface. Optical transmittance spectra were acquired from the same films (now deposited on sapphire substrates). The results corresponding to the 1 nm thick sample (200–1500 nm range) are shown in Figure 2b, exhibiting a high transmittance value over 95% throughout the spectrum. The inset shows the Tauc plot obtained from the transmittance data, with a calculated band gap of 4.7 eV. The high transparency and high optical band gap of the films contribute to their low absorption, making them more effective than traditional doped amorphous Si contacts.

**Figure 2:**
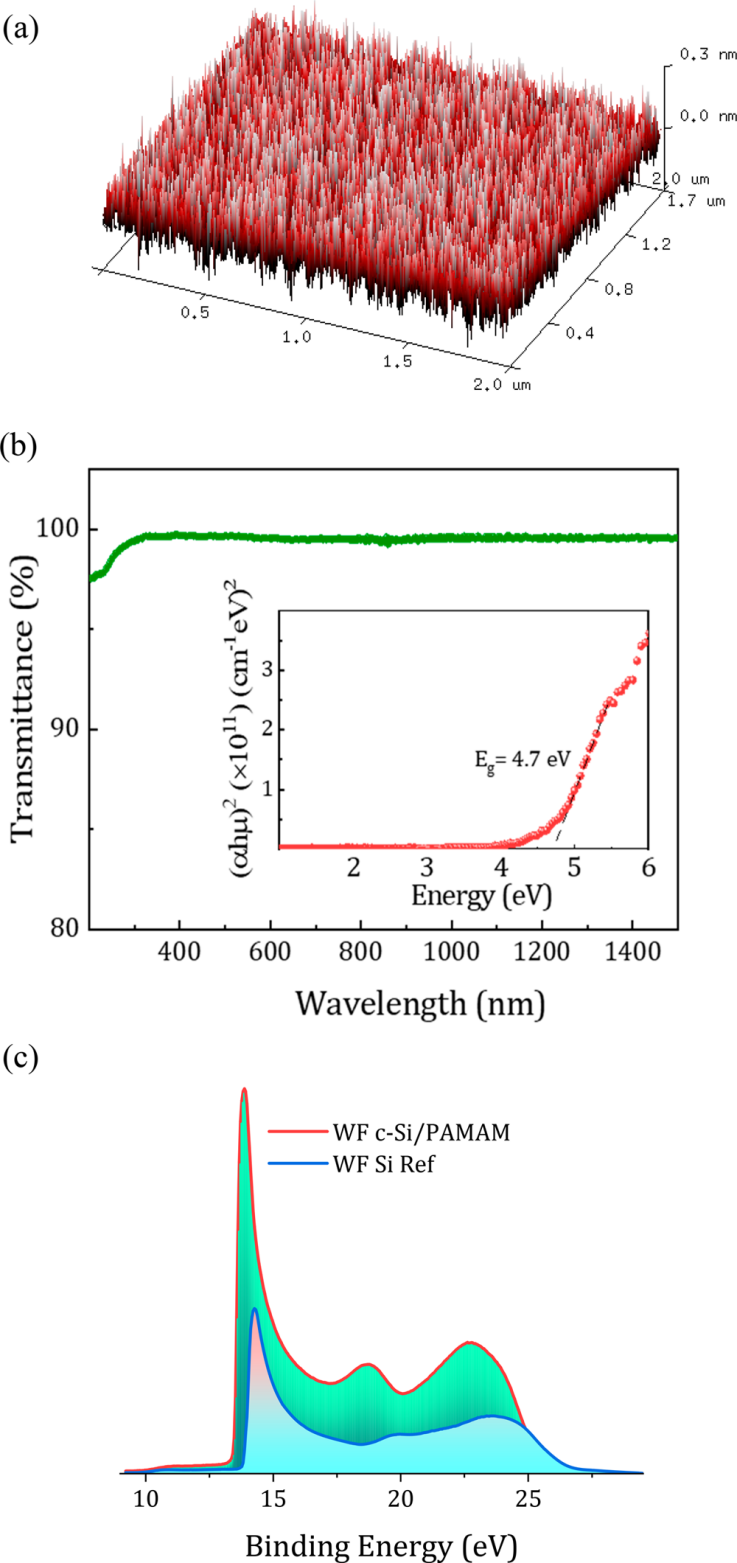
(a) AFM image of the 1 nm thick PAMAM dendrimer film on c-Si (n) with RMS roughness of 0.09 nm. (b) Transmittance spectra of the same films on sapphire substrate, ranging from 200 to 1500 nm. The inset shows the corresponding Tauc plot, showing an optical band gap energy of 4.7 eV. (c) Analysis of the UPS spectra for the same films: work function. The UPS spectra corresponding to the reference c-Si (n) sample are displayed for the sake of comparison.

The chemical analysis of the 1 nm thick PAMAM dendrimer film on the Si substrate was performed using X-ray photoelectron spectroscopy (XPS). Panels a, b, and c of Figure S2 of the Supporting Information display the high-resolution C 1s, N 1s, and O 1s XPS spectra (Supporting Information). N 1s spectra with peaks fitted at 398.7 and 401.1 eV can be attributed to amines and charged amine moieties. The charged amine moieties represent the positively charged N atoms in the PAMAM dendrimer films, with protonated amines occupying 93% of the area in the spectrum.^19−23^ This protonated amine group plays a major role in charge transfer at the Al/PAMAM dendrimer/c-Si interface. It forms a dipole layer at the interface with protonated nitrogen as positive and ethanolate from the counterion condensation as the negative counterpart. This occurrence was proven in our previous work on polyethilimine.^9^

The work function (WF) and valence band position of the film were obtained from ultraviolet (UV) photoelectron spectroscopy (UPS) measurements shown in Figure 2c and Figure S3 of the Supporting Information, respectively. The WF (ϕ) can be calculated using the relation, ϕ = *h*ν – (*E*_cutoff_ – *E*_onset_) – *qV*_bias_, where *h*ν is the incident UV photon energy (21.2 eV) and *E*_cutoff_ is the secondary electron cutoff energy. The thin PAMAM dendrimer film lowers the WF from the reference n-type silicon (4.28 eV) down to 3.69 eV, in good accordance with the expected value.^24,25^ This tuning of the apparent work function at the interface explains the formation of an ohmic contact and the resulting low specific contact resistivity. The work function shift (*q*Δ ≈ 0.6 eV) also indicates the direction of the dipole formation, with the negative end pointing toward the electrode and the positive end pointing toward the silicon substrate. The valence band edge of the c-Si (reference sample) is approximately 1 eV below the Fermi level, which is typical for n-type silicon. On the other hand, the valence band edge of the PAMAM dendrimer film is 2.81 eV below the Fermi level. We may then infer that the film shows a n-type character based on an optical band gap of 4.7 eV and a valence band edge of 2.81 eV. Thus, its n-type character and capability to form an ohmic contact make PAMAM dendrimer a promising candidate as an electron-selective contact in heterojunction solar cells.

The schematic representation of the energy band diagram with the PAMAM dendrimer as the interlayer, proposed on the basis of the determined band gap energy, work function, and valence band edge, is shown in Figure 3a. The PAMAM dendrimer forms a thin dipole intelayer as a result of the protonated amines, as observed from the XPS deconvolved spectra (Figure S2 of the Supporting Information). In turn, this dipole formation between the semiconductor and external electrode leads to the reduction of the apparent metal work function (ϕ′_Al_) with respect to its non-altered value (ϕ_Al_). Consequently, a significant charge transfer from Al to Si takes place that avoids Fermi level pinning at the surface.

**Figure 3:**
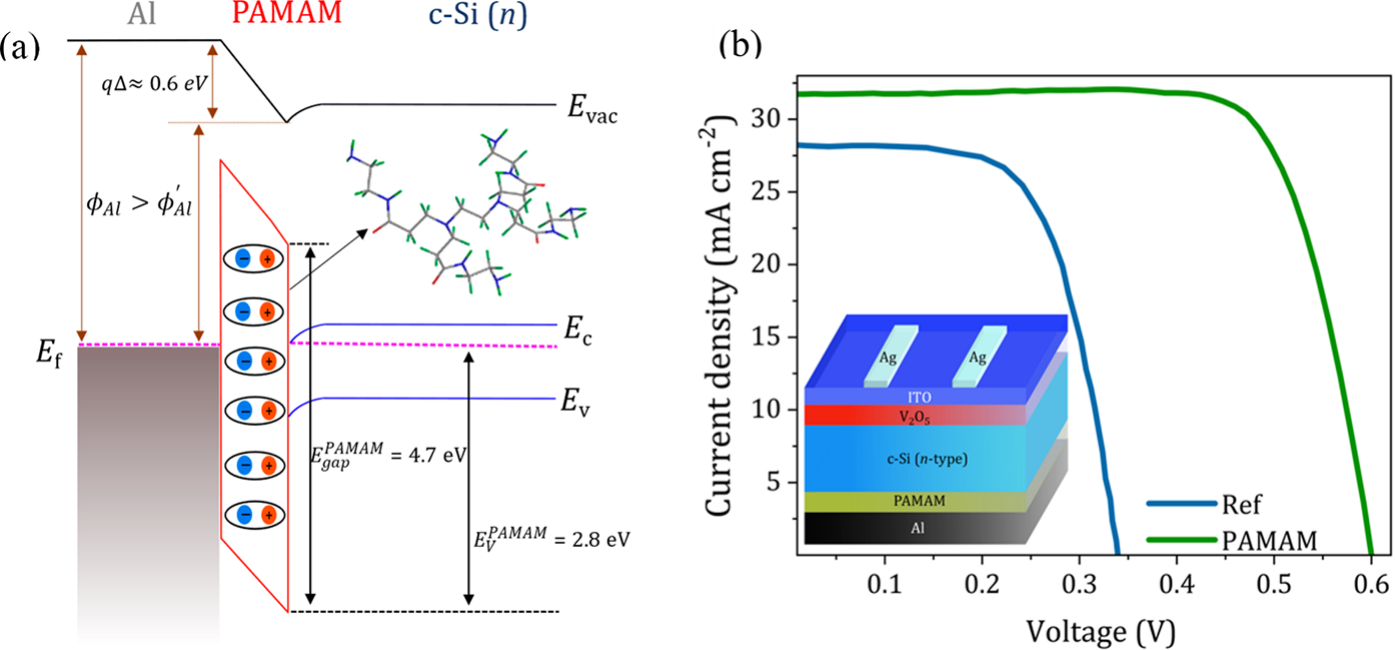
(a) Energy band diagram corresponding to the Si/PAMAM dendrimer/Al heterojunction. (b) *J*–*V* characteristics of the PAMAM dendrimer-based solar cell (green) and the reference device without the PAMAM dendrimer interlayer (blue). The inset shows the architecture of the employed n-type silicon heterojunction cell integrated with a PAMAM dendrimer interlayer as the electron-selective contact.

Finally, as a proof of concept, a PAMAM dendrimer (1 nm)/Al-selective electron contact was integrated into a 1 × 1 cm^2^ doping-free heterojuntion silicon solar cell. V_2_O_5_ was the front hole-selective contact, and the indium-doped tin oxide (ITO) layer was the transparent electrode. The schematic of the fabricated device is shown in the inset of Figure 3b, with an ITO/V_2_O_5_/c-Si/PAMAM dendrimer/Al structure. A reference device was also fabricated without the PAMAM dendrimer to show the superior performance of the solar cell incorporating a PAMAM dendrimer dipole interlayer. Figure 3b shows the current density–voltage (*J*–*V*) curves of both cells measured under 1 sun illumination, and an overview of photovoltaic parameters of the solar cells is shown in Table 1. The evident increase of 260 mV in the open-circuit voltage (*V*_oc_) of the PAMAM dendrimer-based solar cell compared to the reference device can be attributed to the reduced energy barrier at the interface as a result of the formation of an ohmic contact. Additionally, the elimination of Fermi level pinning and the decrease of contact resistance contribute to increase the fill factor (FF). The PAMAM dendrimer-based solar cells have shown an enhanced FF over 76.2%, whereas the reference device exhibits a FF of only 64.4%. The 3.4 mA increase in short-circuit current density (*J*_sc_) in PAMAM dendrimer cells could probably be attributed to electron accumulation at the interface and, hence, the improved surface passivation of silicon. Finally, the PAMAM dendrimer-based solar cells have shown a PCE of 14.5%, more than twice the performance of the reference device.

**Table 1:** Fill Factor (FF), Open-Circuit Voltage (*V*_oc_), Short-Circuit Current Density (*J*_sc_), and Power Conversion Efficiency (PCE) of the Fabricated Solar Cell as well as the Non-containing PAMAM Dendrimer Reference

structure of cells	FF (%)	*V*_oc_ (mV)	*J*_sc_ (mA cm^–2^)	PCE (%)
ITO/V_2_O_5_/c-Si/Al (reference)	64.4	339.8	28.3	6.2
ITO/V_2_O_5_/c-Si/PAMAM/Al	76.2	600.0	31.7	14.5

In conclusion, the obtained results demonstrate that introducing the PAMAM dendrimer as a dipole interlayer between the semiconductor and the metal electrode improves the performance of the solar cell by lowering the metal work function and, thereby, suppressing the Fermi level pinning. As observed, the solar cell devices under study containing the PAMAM dendrimer interlayer at the electron-selective contact have doubled the efficiency with respect to the unmodified reference device. Ultimately, this work demonstrates the promising potential of dipole interlayers in optoelectronic devices whose performance (in this case as a result of photocarrier extraction) can be improved via interface and energy band engineering.

## Experimental Methods

PAMAM dendrimer of the ethylenediamine core, generation 0.0 (G0), dissolved in 20% methanol, with a linear formula NH_2_(CH_2_)_2_NH_2_, was purchased from Sigma-Aldrich. Solutions containing different PAMAM dendrimer concentrations, from 0.1 to 0.001%, were prepared using methanol as the solvent. One-side polished (FZ) n-type c-Si (100) wafers (280 μm thickness, 2 Ω cm resistivity) and sapphire were used as substrates for various electrical and optical studies. Prior to deposition, all Si wafers were treated with 1% HF to achieve an oxide-free silicon surface. The PAMAM dendrimer was spin-coated onto these substrates at 5000 rpm for 30 s and annealed in ambient air on a hot plate for 30 s at 90 °C. The thickness of the PAMAM dendrimer films was measured by ellipsometry, whereas spectroscopic measurements were performed on samples deposited on sapphire glass substrates using UV–visible–near-infrared (NIR) spectrophotometer Lambda 950 (Perkin Elmer, Shelton, CT, U.S.A.). TLM was performed to investigate the specific contact resistance after thermally evaporating Al (300 nm thick) contacts. Morphology studies were carried out using AFM on samples deposited on Si (Bruker Multimode 8 with Nanoscope, Santa Barbara, CA, U.S.A.). XPS and UPS measurements were performed using a Phoibos 150 analyzer (SPECS GmbH, Berlin, Germany). The peaks corresponding to N, C, and O were deconvolved using the Casa XPS software. The work function was then estimated utilizing UPS data from the secondary electron cutoff. For the solar cell fabrication, a PAMAM dendrimer layer was spin-coated onto a (non-texturized) one-side polished n-type silicon wafer followed by ambient air annealing for 30 s. Afterward, 300 nm thick Al was thermally evaporated onto the films as the rear electrode. Atomic-layer-deposited (ALD) V_2_O_5_ was realized as the front hole-selective contact followed by sputtering of the 75 nm thick ITO layer as the transparent electrode.^19^ The active area of 1 × 1 cm^2^ was defined using photolithography, and Ag (1.5 μm) was thermally evaporated using a shadow mask as the top grid. Using a 94041A solar simulator (Newport, Irvine, CA, U.S.A.), the *J*–*V* curves of the cells were measured under standard conditions of 100 mW/cm^2^ and an AM1.5G spectrum. The external quantum efficiency analysis was conducted using the QEX10 equipment (PV Measurements, Point Roberts, WA, U.S.A.).

## Supporting Information Available

(a) Molecular structure of the PAMAM G0 dendrimer and (b) thickness versus concentration plot exhibiting a linear trend (Figure S1), (a–c) deconvolved high-resolution XPS spectra of (a) C 1s, (b) N 1s, and (c) O 1s bonds, for 1 nm thick PAMAM dendrimer films on c-Si (n) (Figure S2), analysis of the UPS spectra of 1 nm films on silicon: valence band determination, with the UPS spectra corresponding to the reference c-Si (n) sample displayed for the sake of comparison (Figure S3), and external quantum efficiency of the PAMAM dendrimer-based solar cell (Figure S4) (PDF)
