# Supplementary material for: Poly(amidoamine) Dendrimer as an Interfacial Dipole Modification in Crystalline Silicon Solar Cells
Source: J Phys Chem Lett. 2023 May 3;14(18):4322–6. doi: 10.1021/acs.jpclett.3c00643 (PMC10184163; doi:10.1021/acs.jpclett.3c00643)
Supplement: Supplementary file 1 — jz3c00643_si_001.pdf [file jz3c00643_si_001.pdf]

Supporting Information

Poly(amidoamine) Dendrimer as an Interfacial Dipole Modification in Crystalline Silicon Solar Cells

[1,2]Thomas Tom*, [3]Eloi Ros*, [1,2]Julian López-Vidrier, [1,2]José Miguel Asensi, [3]Pablo Ortega, [3]Joaquim Puigdollers, [1,2]Joan Bertomeu, [3]Cristóbal Voz**

[1]Departament de Física Aplicada, Universitat de Barcelona (UB), Barcelona 08028, Spain
[2]Institute of Nanoscience and Nanotechnology (IN^{2}UB), Barcelona 08028, Spain
[3]Departament d'Enginyeria Electrònica, Universitat Politècnica de Catalunya (UPC), Barcelona 08034, Spain

** Shared co-first authorship*
***Corresponding author: Cristóbal Voz (cristobal.voz@upc.edu)*

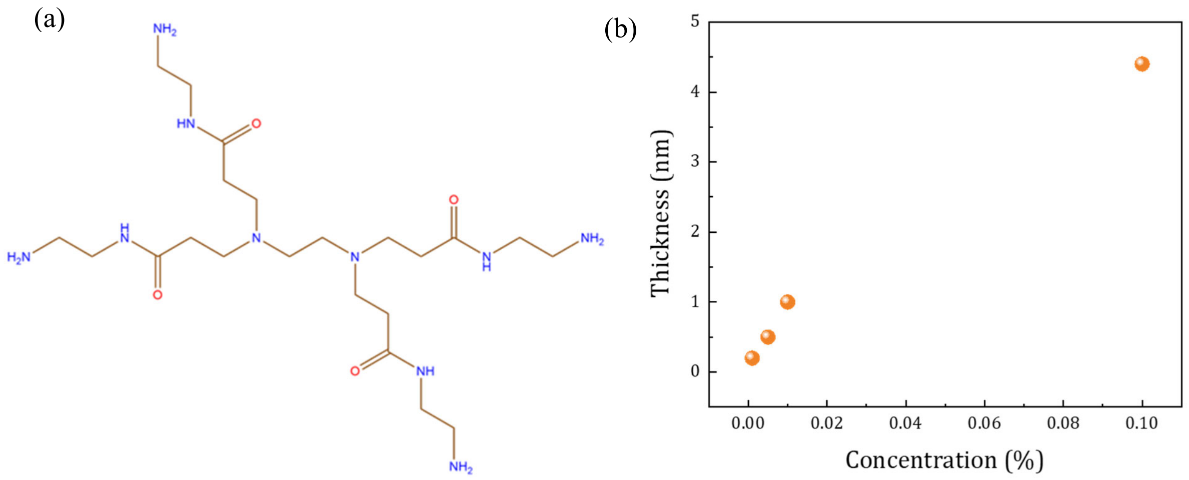

Figure S1. *(a) Molecular structure of the PAMAM G0 dendrimer. (b) Thickness vs. concentration plot exhibiting a linear trend.*

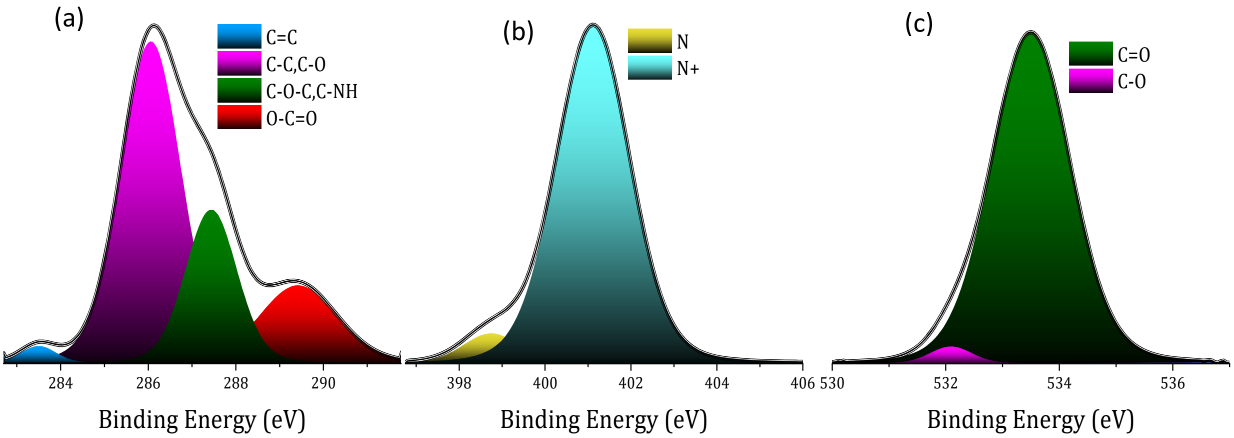

Figure S2. *(a)-(c) Deconvolved high-resolution XPS spectra of C1s (a), N1s (b), and O1s (c) bonds, for1-nm-thick PAMAM dendrimer films on c-Si (n).*

The C1s spectrum is fitted by four peaks at 283.5, 286.1, 287.4, and 289.4 eV. The first peak corresponds to C=C (sp^2 bonded carbons); the second peak can be assigned to C−C and C−O groups; the third peak is ascribed to acetyl C−O−C moieties and carbon-amide C−NH groups; and finally, the fourth peak is attributed to carboxy O−C=O moieties. The O1s spectra could be fitted by two peaks at 532.1 and 533.5 eV, contributions respectively arising from C−O and C=O groups

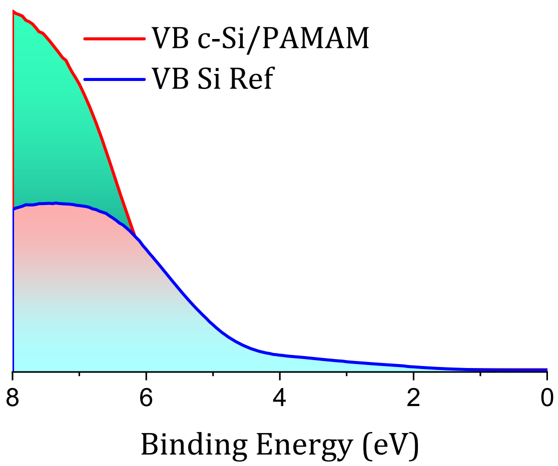

Figure S3. *Analysis of the UPS spectra of 1 nm films on silicon: valence band determination. The UPS spectra corresponding to reference c-Si (n) sample are displayed for the sake of comparison*

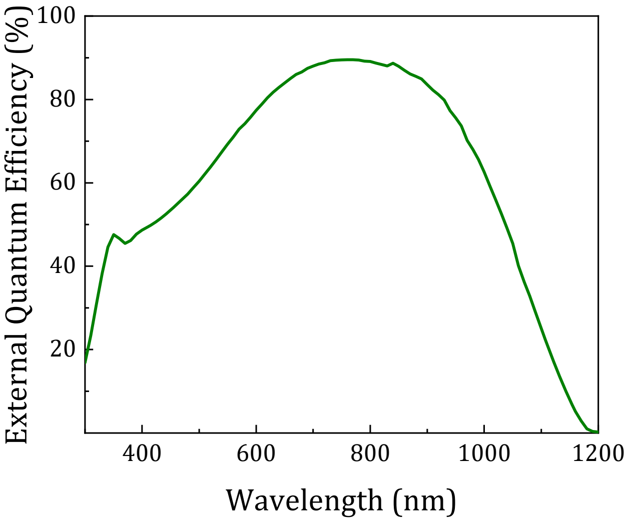

Figure S4. *External quantum efficiency of the PAMAM dendrimer-based solar cell.*
